# Initial Experience With an Amplatzer Cribriform Occluder in Patients With Atrial Septal Defects in Pakistan

**DOI:** 10.7759/cureus.61739

**Published:** 2024-06-05

**Authors:** Saadia Ilyas, Assadullah Khan, Dilnawaz Shah, Zaland A Yousafzai, Qazi Kamran Amin, Saeed Ullah

**Affiliations:** 1 Pediatric Cardiology, Lady Reading Hospital, Peshawar, PAK; 2 Paediatric Cardiology, Lady Reading Hospital, Peshawar, PAK; 3 Cardiology, Lady Reading Hospital, Peshawar, PAK; 4 Medicine, Rehman Medical Institute, Peshawar, PAK; 5 General Medicine, Rehman Medical Institute, Peshawar, PAK

**Keywords:** intervention pediatric cardiology, pediatric cardiology, first experience, atrial septal defect, amplatzer cribriform occluder, : transcatheter closure

## Abstract

Background

Due to their delayed onset of symptoms, atrial septal defects (ASDs) are common congenital cardiac defects that are frequently identified in adulthood. In cases of complicated ASD morphology, transcatheter closure employing devices such as the Amplatzer septal occluder (ASO) presents with difficulties. While the Amplatzer cribriform occluder (ACO) has gained popularity as a specialized option, little is known about its initial use or results, especially in older patients.

Objective

The goal of this study was to describe the early experience with ACO in patients aged 18 to 38 years who had ASDs at a tertiary care hospital in Pakistan, with a focus on the device's efficacy, safety, and viability.

Methods

A total of six cases with ASD who underwent ASD closure with the ACO were retrospectively reviewed at Lady Reading Hospital-Medical Teaching Institution (LRH-MTI), Peshawar, Pakistan. All the required data were obtained from the hospital management information system (HMIS), including patient demographics, defect features, procedure specifics, complications, and outcomes.

Results

Of all patients, 83.3% (n=5) were females and 16.7% (n=1) were males, and the mean age of the group was 27.7 ± 7.9 years. The results of echocardiography showed variation, with a mean fenestrated septum size of 22.4 mm (SD ± 5.4) and a range of device sizes between 18 and 35 mm. The ideal access method for device deployment in every situation was the right femoral vein. There were very few complications; in one instance, a residual shunt necessitated replacing the device. During the six-month follow-up, no complications were found, and all patients were discharged without any problems.

Conclusion

In conclusion, our study indicates that the ACO is a good choice for young adult patients' ASD closure, showing good safety and efficacy. To verify these results and evaluate the long-term functioning of the device, more prospective trials with larger cohorts are required.

## Introduction

Atrial septal defects (ASDs) are the most common congenital heart defect diagnosed in adulthood, as most children and young adults with ASD do not exhibit symptoms, which can cause a delay in identification. As a result, ASD accounts for 25-30% of new diagnoses of congenital heart disease (CHD), making it a prevalent condition identified in adulthood [1]. ASD can be classified into three primary subgroups: secundum ASD, which accounts for the majority (60-75%) of all ASDs, sinus venosus ASD (both superior and inferior), and primum ASD. Percutaneous closure is not feasible in most cases for sinus venosus and primum ASDs, necessitating surgical closure in all cases [2].

For some patients, transcatheter closure of secundum ASDs is a well-recognized substitute for surgical correction. Multifenestrated secundum ASD (F-ASD), which makes up 10% of all ASDs, can be difficult to percutaneously close [3]. Historically, there have been two primary methods for closing ASDs: transcatheter closure with devices, such as the Amplatzer septal occluder (ASO), or surgical intervention. However, there may be challenges when using typical closure devices, especially when the ASD morphology is complicated and includes several defects or irregular shapes. Because of this difficulty, several approaches to ASD closure have been investigated, which has resulted in the creation of specialized tools such as the Amplatzer cribriform occluder (ACO) [4].

Timely intervention to close ASDs in adults is essential as adults with untreated ASD may develop severe arrhythmias, pulmonary vasculopathy, and progressive right ventricular (RV) remodeling and failure. Patients with ASD closure experience improvements in their symptoms as well as regressions in their RV size and pulmonary artery pressure (PAP), all of which are critical to their general health and well-being [5].

ASD cases with multiple fenestrations in Pakistan have traditionally been referred to surgeons, which has resulted in higher expenditures and complications. By using a single device to cover the majority of the septum, the ACO presents a possible alternative to using numerous devices, which can lead to reduced expenses and technical complications. It also has the benefit of not requiring surgery, which has significant dangers, particularly in areas where there is a larger chance of postoperative complications.

Congenital heart defects have a substantial influence on public health in Pakistan, where around 60,000 children are born with CHD each year [6]. The implementation of the ACO at Lady Reading Hospital represents a significant breakthrough in the treatment of multifenestrated ASDs. Its early use and results in this regard, however, have not been investigated in Pakistan. This study intends to provide our initial experiences with the device in young adult patients aged 18 to 38 years in a Peshawar tertiary care hospital. We seek to improve knowledge of transcatheter ASD closure and provide guidance for clinical practices through our assessment of the device's viability, safety, and effectiveness.

## Materials and methods

Six ASD closures at Lady Reading Hospital - Medical Teaching Institution (LRH-MTI), Peshawar, Pakistan, using the ACO were studied in this retrospective review. The procedures were performed between March 2022 and March 2023. The Hospital Management Information System (HMIS) provided the data for the study, which concentrated on patient demographics, defect features, procedure specifics, complications, and outcomes.

Patient selection

Patients who had fenestrated ASDs (F-ASDs) and underwent device closure at LRH-MTI Peshawar between March 2022 and March 2023 using the ACO were included in the study.

Data collection

Age and gender were among the demographic details that were noted for every patient. The HMIS was used to retrieve specific echocardiographic results, such as the collective sizes of F-ASDs, pulmonary arterial hypertension (PAH), and RV overload. Information was also recorded about the characteristics of the device, the procedural strategy (such as using the femoral vein), any intraprocedural complications, and the results of the procedure.

Ethical considerations

All patients participating in the study gave their informed consent prior to data collection. The LRH-MTI Institutional Review Board granted ethical permission for the retrospective review, guaranteeing adherence to principles of ethics.

Data analysis

The demographics, defect morphology, procedure specifics, complications, and results of the six cases were summarized using descriptive statistics. When applicable, means with standard deviations or medians with interquartile ranges were used to display continuous variables. Frequencies and percentages were used to express categorical variables.

Limitations

Retrospective analyses have intrinsic limitations that apply to this study, such as inadequate data documentation and probable selection bias. Furthermore, the findings' generalizability might be restricted by the limited sample size of only six cases.

Confidentiality

Throughout the course of the study, patient privacy was protected by implementing data anonymization techniques and rigorously maintaining patient confidentiality.

Follow-up

For a minimum of six months following the procedure, all patients underwent routine follow-up at one-month intervals with transthoracic echocardiograms and 12-lead electrocardiograms.

## Results

In this retrospective study, six cases (mean age of 27.7±7.9 years) at LRH-MTI Peshawar underwent ASD closure using the ACO. Of the cohort, 83.3% (n=5) were females and 16.7% (n=1) were males.

The results of the echocardiogram showed a variety of ASD morphologies, such as several defects with RV overload and left-to-right shunting, two defects with a septum in between, and multiple small ASDs. The average size of the F-ASD septum was 22.4 mm (SD ± 5.4), with a range of 14 to 28 mm. The ACO’s dimensions ranged from 18 to 35 mm.

As for the procedural approach, in every case, the right femoral vein was accessed for the deployment of the device using a transfemoral technique. For monitoring purposes, in one patient, 16.7% (n=1), access to the right femoral artery was adopted.

All patients were discharged without incident. During the six-month follow-up, no complications were noted, demonstrating that the ACO was effective in closing ASDs. In our study group, complications were rare; only one case, 16.7% (n=1), required device replacement due to a residual shunt. There were no additional documented intraoperative or post-procedural complications nor any cases of device embolization or malposition. A summary of patient characteristics and procedural details is provided in Table 1.

**Table 1:** Summary of patient characteristics and procedural details ASD, atrial septal defect; Lt, left; Rt, right; RV, right ventricle

Case No.	Age (in Years)	Gender	Echocardiography Findings	Fenestrated Septum Size	Device Size	Approach	Complications	Outcome	Follow-up at 6 Months
1.	27	Female	Multiple ASD, Lt to Rt shunt, RV overload	24.6 mm	30 mm	Right femoral vein	None	Discharged	Uneventful
2.	38	Female	Multiple ASD, Lt to Rt shunt, RV overload	20.5 mm	25 mm replaced with 30 mm	Right femoral vein	Residual shunt, device replaced	Discharged	Uneventful
3.	32	Female	Two defects with septum in between	28 mm	35 mm	Right femoral vein	None	Discharged	Uneventful
4.	18	Female	Multiple small ASDs	18.8 mm	25 mm	Right femoral vein	None	Discharged	Uneventful
5.	29	Female	Multiple small ASDs	26.5 mm	30 mm	Right femoral artery for monitoring	None	Discharged	Uneventful
6.	22	Male	Three small defects	14 mm	18 mm	Right femoral vein	None	Discharged	Uneventful

Intravenous sedation and local anesthesia were used for all procedures. None of the instances required intubation. Intravenous heparin administered at 100 units/kg was used to maintain anticoagulation during the procedure. The median fluoroscopy time was 7.8 minutes (range: 3.5 to 32.1 minutes).

## Discussion

One of the most prevalent congenital cardiac anomalies in adults is ASD. Patients with ASD may present at any age, and of those who have the secundum type, 65-75% are female; in contrast, the gender distribution for those with the sinus venosus and ostium primum types is equal [7]. A 2011 study from Nigeria examining adult patients with ASDs revealed that nine were males and 23 were females. This is in accordance with our study, in which 83.3% of the patients were females [8]. With 25-30% of cases, ASD is the most frequent congenital heart disease in adults, and 80% of ASDs are secundum ASDs, which are found in the fossa ovalis. When possible, device closure is the recommended course of action [1].

ASDs have historically been linked to a variety of complications that mostly impact the right side of the heart, such as thromboembolism, arrhythmias, right heart failure, and, in some cases, PAH. The left heart is similarly affected by the pathophysiology of ASDs, mostly as a result of volume unloading and negative ventricular-ventricular interaction [1,9].

Improved cardiac output, improved functional class, increased exercise capacity, and reverse RV remodeling are all associated with ASD closure in most patients, regardless of age [9]. Following ASD closure, ventricular remodeling entails a reduction in RV size and PAP; patients who have less functional impairment and lower PAP levels tend to have better outcomes. Moreover, better exercise ability and long-term cardiovascular advantages are linked to the closure of ASD in adults [10]. Following repair of ASDs by surgical or catheter-based procedures, mortality rates have improved in current populations, including the elderly. These results are consistent with what has been seen in the general population over mid- to longer-term follow-up periods [11-13].

ASD catheter closure techniques and equipment have significantly improved over time. With very low complication rates and a shorter hospital stay and recovery compared to surgery and avoidance of cardiopulmonary bypass, device closure is the therapy of choice for the majority of patients with a secundum ASD [1,14].

In 1974, King and Mills deployed the first transvenous device to seal an ASD. Thirteen animals with experimentally punctured ASDs were subjected to an experimental device. Successful closure was attained by five animals [15,16]. Currently, the Gore cardioform septal occluder, Amplatzer cribriform, and ASO are the three devices that the U.S. Food and Drug Administration (FDA) has approved for the closure of ASDs in the United States.

For the closure of multifenestrated secundum-type ASDs, the Amplatzer™ multi-fenestrated septal occluder, also known as the cribriform occluder, has received approval. The left and right atrial discs of the device are identical in size and have a short central waist, which sets it apart from the ASO in terms of design. The device is available in 18-, 25-, 30-, and 35-mm diameters, depending on the size of the disk. Since the cribriform occluder has a short central waist, it is not self-centering like the ASO. Indications include patients exhibiting signs of RV dysfunction and cribriform ASDs [17].

A study on the technical viability of closing F-ASDs with the ACO was carried out by Numan et al. mostly in the pediatric population. With transcatheter closure of secundum ASD, their small sample of sixteen patients had an 81.2% success rate; 76.9% of them achieved complete closure by the following day, and the remaining patients achieved closure within six to 12 months [18]. The treatment resulted in a statistically significant reduction in RV diastolic pressure without apparent problems such as stroke, conduction anomalies, or fatalities, suggesting the device's safety and efficacy for F-ASD closure. Transcatheter closure was used in a different 2016 trial to treat eight patients with F-ASDs and accompanying pulmonary hypertension and/or ventricular dysfunction [19]. This procedure improved the patients' symptoms and exercise tolerance without causing problems with the device, infections, or strokes. The study revealed that even partial closure of ASDs might be safe and advantageous in patients with F-ASDs and ventricular dysfunction or pulmonary hypertension.

In our research, we found that the ACO offered a number of benefits, such as the capacity to cover the majority of the septum with a single device, eliminating the need for numerous devices, and cutting down on the complexity and expense of the procedure. The device's adaptability and versatility during the process were further demonstrated in a scenario we encountered when a larger device was needed because of a residual flow. Although there were only six cases in the initial experience, our data indicate positive outcomes, indicating that the ACO may have usefulness in clinical practice. These findings highlight the need to share our first experience with other centers to stimulate device adoption and further study in other patient demographics and contexts.

## Conclusions

Our research highlights the ACO's potential as a secure and reliable substitute for ASD closure in older patients. To evaluate the long-term benefits of this novel device in the local community and support our findings, more prospective studies with larger cohorts and longer follow-up times are needed.
